# Benefits of using digital thoracic drainage systems for post-operative treatment in pediatric populations: personal experience and review of literature

**DOI:** 10.3389/fped.2023.1280834

**Published:** 2023-10-11

**Authors:** Simone Frediani, Giorgia Romano, Valerio Pardi, Ivan Pietro Aloi, Arianna Bertocchini, Antonella Accinni, Angelo Zarfati, Alessandro Inserra

**Affiliations:** General and Thoracic Pediatric Surgery Unit, Bambino Gesù Children’s Hospital, IRCCS, Rome, Italy

**Keywords:** pleurevac, thoracic drainage, children, chest tube, digital chest drainage

## Abstract

**Introduction:**

The digital chest drainage monitoring system (Medela Thopaz+), unlike analogical systems, reliably regulates the pressure applied to the patient's chest and digitally and silently monitors critical therapeutic indicators (volume of fluid and/or drained air). Its use in adulthood has been widely described, but there is still little experience in the pediatric field. The aim of this study is to test this new device in the pediatric population.

**Materials and methods:**

We conducted a retrospective study of 160 patients undergoing chest surgery at our Hospital. These patients were divided into 82 treated with the Thopaz system in the period from January 2021 to April 2023 and 78 in whom Pleurevac, had been used in the time period from January 2020 to April 2023.

**Results:**

The average age of patients was 10.45 years (range: 3.1–17.2) for the Thopaz Group and 10.71 years for Pleurevac Group. The groups were homogeneus also by weight and type of intervention. The device was held in place for 10.64 days (mean) for Thopaz Group, compared to 16.87 days (mean) for Pleurevac Group (*p* < 0.05). The median number of postoperative x-rays before the closure of the chest tube was 4.29 in the digital drainage group compared to 8.41 in the traditional draining group (*p* < 0.05).

**Conclusions:**

The digital chest monitoring device provides objective measurement, allows for rapid patient mobilization (with good pain control and increased compliance). In addition, the use of Thopaz in the paediatric population seems to be safe (there is no statistically significant difference in terms of complications such as prolonged air leaks and pneumothorax after the chest tube closure) and potentially beneficial.

## Introduction

**Level of Evidence:** IV

When there is an abnormal amount of air or fluid in the pleural cavity, thoracic drainage is used to help it leak out, allowing the lungs to fully expand again and the normal breathing mechanism to be restored.

New digital chest drainage systems are changing the management of the chest tube. These new devices provide real-time information and offer the opportunity to follow what happened during the period when there was no direct observer in front of the drainage, facilitating the delicate management of paediatric patients carrying a chest tube in the immediate post-operative period. The main goal is to increase safety when deciding whether to maintain the drainage, so as not to extend its permanence longer than necessary or remove it too early with the risk of complications.

In particular, our analysis is based on the comparison between analogical and digital systems and the examination of the possible advantages and disadvantages of using Thopaz:

- the quantification and recording of air leaks and the recognition of minimum active leaks by distinguishing them from apparent leaks due to a pleural space effect;
- the facilitation of decision-making at the time of removal of the device;
- reduce the duration of treatment for patients with a chest tube;
- reduce the duration of the presence of chest tube.
- the possibility of early mobilization of the patient from the bed regardless of the presence of drainage

## Materials and methods

We conducted a retrospective study. All patients undergoing thoracic surgery from January 2021 to April 2023 in which a digital thoracic drainage system (Thopaz Chest Drain System, Medela, Switzerland) was placed were included in this study. This group was compared to a historical cohort of patients homogeneous by age, weight and type of intervention, operated from 2020 to 2023 with placement of a chest drainage and use of the traditional Pleur-evac aspiration system (Teleflex, MINI SAHARA, Pleur-evac and Sahara are registered trademarks of Teleflex Incorporated or its affiliates).

We reviewed the clinical records of the 160 patients identified by this study by analysing the following parameters:

- the average duration of the chest tube stay (days),
- the average duration of the drainage (days),
- the appearance of pneumothorax after the closure of the chest tube,
- the presence of prolonged air leaks and/or pleural spill.

The basic characteristics of the patients were collected such as: gender, age and weight at the time of the intervention, pathology (the reason for hospitalization), the side involved, the date of acceptance and discharge. For each patient, the type of surgery performed, thoracotomy or thoracoscopic approach, the size of the chest tube used, type of aspiration applied, and the number of chest x-rays performed before the chest tube was removed were also conducted. The choice of the size of the thoracic tube and the place of insertion was left to the surgeon. The removal of the chest tube was possible after a complete pulmonary re-expansion was determined, only after obtaining a chest x-ray with a closed chest tube for 24 h. The presence of “air leak” has been if, after closing the chest tube, it has become necessary to re-open the chest tube for spill collection or for prolonged air leaks. The digital chest drainage system used was the Thopaz Chest Drain System, Medela, Switzerland, placed in 82 patients, while the traditional aspiration system, used in the 78 patients in the control group, was Pleur-evac. Our analysis later focused on comparing the data obtained from the use of the two different drainage systems following the same surgical procedure. We calculated the average duration of the presence of the chest tube (days), the average period of length of stay (days), the postoperative complications associated with air leaks, i.e., prolonged air leak (“air leak”) and/or pleural spill and appearance of pneumothorax after the chest tube closure, following 4 types of interventions:

1) Thoracic drainage
2) The bullectomy
3) Pulmonary resection (lobectomy or segmentectomy)
4) Removal of thoracic mass without pulmonary resection.

We also compared the degree of satisfaction of patients and healthcare staff.

Dichotomous variables were analyzed using Fisher's exact test. Data were also correlated by Pearson's coefficient, and 95% confidence intervals were calculated. Statistical tests were performed using GraphPad Prism for Windows (GraphPad Software, San Diego, CA; http://www.graphpad.com). A VAS scale (Visual Analogue Scale) was used to assess the satisfaction of healthcare personnel and patient tolerability.

## Results

The digital drainage system was used in 82 patients, 55 males and 27 females. The average age was 10.45 years, and the median weight was 39.55 kg. The most common operation was thoracic positioning of a chest tube (drainage), which was done on 29 patients. This was followed by bullectomy, which was done on 9 patients, pulmonary resection, which was done on 23 patients, and removal of the chest mass without removing the lung, which was done on 14 patients. The procedures were carried out with a thoracoscopic approach in 27 patients and a thoracotomy in 36. The historical cohort included 78 patients: 49 males and 29 females. The median age was 10.71 years, and the average weight was 38.43 kg. 25 patients were recovered. In 21, the thoracic positioning of a chest tube (drainage), in 18, the removal of the chest mass without pulmonary resection, and in 12, the bullectomy. The interventions were carried out for thoracoscopy in 31 patients and for thoracotomy in 27 patients. The two groups were comparable by age, weight, diagnosis, and type of the procedure (Table 1).

**Table 1:** Characteristics of the patients.

	Thopaz	Pleurevac	*P*
Patients	82	78	
Age (years)	10.45^a^	10.71^a^	0.878
Weight (Kg)	39.55^a^	38.43^a^	0.756
Sex (M:F)	55/27	49/29	
Thoracotomy	36	27	
Thoracoscopy	27	31	
Drainage	16	18	
Laparotomy with diaphragm's opening	3	2	
Side Right/Left	43/35	52/23	
Bilateral	4	3	
Days with thoracic drainage	10.64^a^	16.87^a^	0.009
Chest X-ray before drainage closure (*N*)	4.29^a^	8.41^a^	0.021
Lenght of stay (dd)	21.55^a^	29.3^a^	0.012
Prolonged air-leak	19.5% (16)	17.9% (14)	0.841
Pneumothorax after drainage's closure	4.82% (4)	7.6% (6)	0.527

^a^ Value are means

The median duration of days of chest tube reception was 10.64 days in the digital drainage group compared to 16.87 days in the traditional drainage group (*p* < 0.05). The median number of postoperative x-rays before the closure of the chest tube was 4.29 in the digital drainage group compared to 8.41 in the traditional draining group (*p* < 0.05): the information provided by the digital device on the air flow allowed to reduce ionizing radiation. The median degeneration in the digital drainage group was 21.55 days compared to 29.3 days in the traditional draining group (*p* < 0.05). In the digital drainage system group, 20 patients experienced postoperative complications related to air leakage: 16 (19.5%) patients experienced prolonged air leaks, and 4 (4.88%) patients developed pneumothorax after the chest tube closure. In the historical cohort, 14 (17.9%) patients experienced persistent air loss and 6 (7.69%) experienced a pneumothorax after thoracic tube closure (*p* > 0.05) (Table 1).

The analysis of each type of intervention showed that in all four assessed procedures, thoracic positioning of a chest tube (Table 2), bullectomy (Table 2), pulmonary resection (Table 2) and thoracic mass removal (Table 2), the use of the digital system allowed to reduce the duration of drainage, the number of post-operative chest Xrays and the days of hospitalization (*p* < 0,05). No statistically significant differences were detected between the two groups of patients treated with analogic devices compared to those in which the digital system was used in the incidence of complications, including prolonged air loss and outpouring and the appearance of remote pneumothorax (*p* > 0.05). We evaluated the degree of satisfaction of patients and healthcare staff, surgeons and nurses, about their experience with the use of the Thopaz system; our survey revealed higher satisfaction from the digital system vs. the analogic system (*p* < 0,05). (Table 3) Of the nine surgeons interviewed, eight expressed their opinion in favour of the digital system, which was considered safer at the time the chest tube was closed than the traditional system.

**Table 2:** The analysis of each type of intervention.

Drainage	Thopaz (29)	Pleurevac (21)	*P*
Days with thoracic drainage	15.57^a^ (5–96)	19.79^a^ (2–51)	0.014
Chest X-ray before drainage closure (*N*)	6.89^a^ (1–42)	8.9^a^ (1–29)	0.032
Lenght of stay (dd)	34.45^a^ (6–161)	38.26^a^ (3–104)	0.048
Prolonged air-leak	37.9% (11)	29% (6)	0.5565
Pneumothorax after drainage’s closure	6.9% (2)	15% (3)	0.6378
Lung resection	Thopaz (9)	Pleurevac (12)	*P*
Days with thoracic drainage	8.11^a^ (4–14)	16.68^a^ (23–8)	0.025
Chest X-ray before drainage closure (*N*)	2^a^ (1–4)	4.1^a^ (2–17)	0.008
Lenght of stay (dd)	10.11^a^ (5–17)	22.43^a^ (10–41)	0.002
Prolonged air-leak	22% (2)	25% (3)	0.398
Pneumothorax after drainage’s closure	11 % (1)	17% (2)	0.590
Resezioni	Thopaz (23)	Pleurevac (25)	*P*
Days with thoracic drainage	7.35^a^ (5–13)	15.67^a^ (9–21)	0.0098
Chest X-ray before drainage closure (*N*)	2.52^a^ (1–7)	5.71^a^ (1–12)	0.031
Lenght of stay (dd)	12.57^a^ (6–66)	22.43^a^ (10–42)	0.0002
Prolonged air-leak	13% (3)	16% (4)	0.9999
Pneumothorax after drainage’s closure	0% (0)	4% (1)	0.9999
Chest mass removal	Thopaz (14)	Pleurevac (18)	*P*
Days with thoracic drainage	6.29^a^ (3–11)	12.32^a^ (11–23)	0.0249
Chest X-ray before drainage closure (*N*)	2.78^a^ (1–6)	6.23^a^ (1–13)	0.0396
Lenght of stay (dd)	16^a^ (6–45)	21.3 (11–39)	0.0051
Prolonged air-leak	7% (1)	6% (1)	0.9999
Pneumothorax after drainage’s closure	0% (0)	0% (0)	0.9999

^a^ Value are means

**Table 3:** Evaluation of the level of satisfaction of patients and healthcare personnel.

	Pleurevac	Thopaz	*P*
Surgeons rating	7^a^	9.3	0.0002
Nurse rating	6.7^a^	8.6^a^	0.0013
Patients rating	6.3^a^	9.1^a^	0.0001

^a^ Value are means

## Discussion

Adequate drainage of the pleural cavity is one of the most important aspects of postoperative chest surgery, and identifying the right time for its removal is often a controversial issue, even among the most experienced surgeons. With the use of traditional drainage tubes, in fact, the assessment of the presence of air leaks is linked to the subjective interpretation of the operator who observes any “bubbles” in the collection chamber, depending therefore on the level of experience of the doctor (1–3).

Despite the development of classification systems for air leaks, there is frequently disagreement among observers not only about the extent or clinical significance of a loss but sometimes also about its presence or not. When the uncertainty arising from the use of a traditional system persists, it inevitably prolongs the length of stay, resulting in greater use of resources and time (4, 5).

The problem of low sensitivity and extreme interindividual variability resulting from this method has led to the need to develop more sophisticated digital systems that can ensure the maintenance of a regulated negative pressure, provide an objective and systematic assessment of air leaks, and standardize the timing of drainage removal (6–9).

The use of the latest digital drainage systems has already proven effective in postoperative chest surgery in adults (Table 4) (10–13); the National Institute for Health and Care Excellence (NICE), an independent British organization dedicated to promoting national and international guidelines for good clinical practice, recommends the adoption of Thopaz+ for the management of chest drainages in patients undergoing lung resection (14–18) and pneumothorax (19–22). The NICE recommendations are based on evidence from studies, clinical experts, and local public and private authorities and are the result of rigorous, objective, and independent assessments. The recommendations for use and the benefits associated with the use of Thopaz+ for thoracic drainage are derived from the analysis of 13 randomized controlled and comparative studies (total number of patients *n* = 1,632), of which eleven were dedicated to the use of Thopaz+ after pulmonary resection and two included patients with pneumothorax.

**Table 4:** Literature review of the adult population.

	Article (title, author)	Pubblication year	Journal	Number of patients
1	**Digital versus analogue chest drainage system in patients with primary spontaneous pneumothorax: a randomized controlled trial*;*** *Dieuwertje Ruigrok, Peter W. A. Kunst, Marielle M. J. Blacha, Ben Tomlow, Jacobine W. Herbrink, Eva J. Japenga, Wim Boersma, Paul Bresser, Ivo van der Lee and Kris Mooren*	2020	BMC pulmonary medicine	52
2	**Use of Thopaz in Patients of Empyema Thoracis Undergoing Decortication;** *Mohd. Shahnawaz Alam, Mohd. Azam Hasee, Mohd. Aslam, Mohd. Hanif Beg*	2020	Lung India	50
3	**Comparison of clinical utility between digital and analog drainage systems in patients with spontaneous pneumothorax;** *Shota Yagi, Hideki Miwa, Masato Kono, Shin Ikeda, Tomo Tsunoda, Ryutaro Hirama, Masayuki Watanuki, Yuiko Oshima, Akari Tsutsumi, Yoshihiro Miki, Dai Hashimoto, Hidenori Nakamura*	2022	Respiratory Investigation	64
4	**Clinical application of a digital thoracic drainage system for objectifying and quantifying air leak versus the traditional vacuum system: a retrospective observational study;** *Song Am Lee, Jun Seok Kim, Hyun Keun Chee, Jae Joon Hwang, Michael Ji, Yo Han Kim, Hyeong Ju Moon, Woo Surng Lee*	2021	Journal of thoracic disease	50
5	**Bubbles-in-the-chamber vs digital screen in chest drainage: A blind analysis of compared postoperative air leaks evaluation;** *Giuseppe Marulli, Debora Brascia, Giulia De Iaco, Giovanni Maria Comacchio, Giuseppe Natale, Mario Nosotti, Paolo Mendogni, Sara Pieropan, Camillo Lopez, Gaetano Di Rienzo, Luigi Gaetano Andriolo, Federico Rea*	2020	Heart & Lung	117
6	**Randomized trial of digital versus analog pleural drainage in patients with or without a pulmonary air leak after lung resection;** *Sebastien Gilbert, Anna L. McGuire, Sonam Maghera, Sudhir R. Sundaresan, Andrew J. Seely, Donna E. Maziak, Farid M. Shamji, and P. James Villeneuve*	2015	Journal of Thoracic & Cardiovascular Surgery	172
7	**Regulated tailored suction vs regulated seal: a prospective randomized trial on air leak duration;** *Alessandro Brunelli, Michele Salati, Cecilia Pompili, Majed Refai and Armando Sabbatini*	2012	European Journal of Cardio-Thoracic Surgery	100
8	**Efficacy assessment of the drainage with permanent airflow measurement in the treatment of pneumothorax with air leak;** *Slawomir Jablonski, Marian Brocki, Marcin Wawrzycki, Jacek Arkadiusz Smigielski, Marcin Kozakiewicz*	2013	The Thoracic and cardiovascular surgeon	60
9	**Electronic versus traditional chest tube drainage following lobectomy: a randomized trial;** *Marike Lijkendijk, Peter B. Licht and Kirsten Neckelmann*	2015	European journal of cardio-thoracic surgery	105
10	**Postoperative chest tube management: snapshot of German diversity;** *Albert Linder, Clemens Ertner, Volker Steger, antje Messerschmidt, Johannes Merk, Inez Cregan, Jürgen Timm and Thorsten Walles*	2012	Interactive cardiovascular and thoracic surgery	79
11	**The implementation of a digital chest drainage system significantly reduces complication rates after lobectomy – a randomized clinical trial;** *Tomasz Marjański, Adam Sternau, Witold Rzyman*	2013	Polish Journal of Thoracic and Cardiovascular Surgery	64
12	**The benefits of digital air leak assessment after pulmonary resection: prospective and comparative study;** *José M. Mier, Laureano Molins y Juan J. Fibla*	2010	Cirugía española	75
13	**Digital Drainage System Reduces Hospitalization After Video-Assisted Thoracoscopic Surgery Lung Resection;** *Daniel L. Miller, MD, Gerald A. Helms, MD, and William R. Mayfield, MD*	2016	The Annals of thoracic surgery	108
14	**Impact of the learning curve in the use of a novel electronic chest drainage system after pulmonary lobectomy: a case-matched analysis on the duration of chest tube usage;** *Cecilia Pompili, Alessandro Brunelli, Michele Salati, Majed Refai, Armando Sabbatini*	2011	Interactive cardiovascular and thoracic surgery	102
15	**Multicenter international randomized comparison of objective and subjective outcomes between electronic and traditional chest drainage systems;** *Cecilia Pompili, Frank Detterbeck, Kostas Papagiannopoulos, Alan Sihoe, MB BChir, FRCSEd(CTh), Kostas Vachlas, Mark W. Maxfield, Henry C. Lim and Alessandro Brunelli*	2014	The Annals of thoracic surgery	381
16	**Clinical Evaluation and Outcomes of Digital Chest Drainage after Lung Resection;** *Fumihiro Shoji, Shinkichi Takamori, Takaki Akamine, Gouji Toyokawa, Yosuke Morodomi; Tatsuro Okamoto, Yoshihiko Maehara*	2016	Annals of Thoracic and Cardiovascular Surgery	121
17	**A pilot study of a digital drainage system in pneumothorax;** *Georgia Tunnicliffe, Adrian Draper*	2014	BMJ open respiratory research	13
18	**Postoperative Air Leaks After Lung Surgery: Predictors, Intraoperative Techniques, and Postoperative Management;** *Travis C. Geraci, Stephanie H. Chang, Savan K. Shah, Amie Kent, Robert J. Cerfolio*	2021	Thoracic surgery clinics	
19	**What is the optimal level of suction on digital chest drainage devices following pulmonary lobectomy?;** *Marlene Fromm Sørensen, Bo Laksa ´foss Holbek, Rene ´ Horsleben Petersen and Thomas Decker Christensen*	2021	Interactive cardiovascular and thoracic surgery	367
20	**Early experience with the Thopaz+ chest drainage system – is this a new era in the management of post-cardiotomy bleeding?;** *Karolina Pawelkowska, Stanislaw Bartus, Robert Sobczynski, Michal Medrzycki, Grzegorz Grudzien, Grzegorz Filip, Bartosz Cierpikowski, Krzysztof Bartus, Boguslaw Kapelak*	2021	Polish journal of cardio-thoracic surgery	42
21	**Novel, digital, chest drainage system in cardiac surgery;** *Luca Barozzi, Livio San Biagio, Matteo Meneguzzi, Delphine S. Courvoisier, Beat H. Walpoth, Giuseppe Faggian*	2020	Journal of cardiac surgery	120
22	**Multicenter randomized controlled trial comparing digital and traditional chest drain in a VATS pulmonary lobectomy cohort: interim analysis;** *Paolo Mendogni, Davide Tosi, Giuseppe Marulli, Giovanni Maria Comacchio, Sara Pieropan, Veronica Rossi, Debora Brascia, Luigi Gaetano Andriolo, Giovanna Imbriglio, Gianluca Bonitta, Camillo Lopez, Federico Rea and Mario Nosotti*	2021	Journal of Cardiothoracic Surgery	231
23	**The benefits of digital drainage system versus traditional drainage system after robotic-assisted pulmonary lobectomy;** *Kristina Jacobsen, Steven Talbert, Joseph H. Boyer*	2019	Journal of Thoracic Disease	182
24	**A Systematic Review of Digital vs Analog Drainage for Air Leak After Surgical Resection or Spontaneous Pneumothorax;** *Fadi Aldaghlawi, Jonathan S. Kurman, Jason A. Lilly, D. Kyle Hogarth, Jessica Donington, Mark K. Ferguson and Septimiu D. Murgu*	2020	Chest	
25	**Complications after Chest Tube Removal and Reinterventions in Patients with Digital Drainage Systems;** *Yi-Ying Lee, Po-Kuei Hsu, Chien-Sheng Huang, Yu-Chung Wu and Han-Shui Hsu*	2019	Journal of Clinical Medicine	497
26	**Work in progress report of a multicentre retrospective observational study to evaluate the association between the airflows and the intrapleural pressures digitally recorded after video-assisted lobectomy;** *Luca Bertolaccini, Andrea Viti, Pietro Bertoglio, Andrea Imperatori, Angelo Morelli, Francesco Zaraca, Lorenzo Spaggiari and Roberto Crisci*	2021	Interactive cardiovascular and thoracic surgery	76
27	**Digital chest drainage systems are beneficial for robotic-assisted lung resections;** *Christopher Lau, Sebron Harrison*	2020	Journal of thoracic disease	

When compared to the standard thoracic drainage system with wall aspiration, drainage times and hospital stays were much shorter when Thopaz+ was used. However, there were no statistically significant differences in the rates of re-insertion of thoracic drainage after a spontaneous pneumothorax.

Results from studies in adults:

Thopaz+ leads to clinical improvements in patients who need chest drainage after pulmonary resection or due to pneumothorax, with a significant reduction in drain time and duration of hospitalization (23–25). Other advantages include standardization of decision-making, improved patient safety and satisfaction, as well as staff confidence, compared to conventional breast drainage systems with wall aspiration (26–28).
The potential for savings resulting from the adoption of Thopaz+ for the management of chest drainage is £111 per patient undergoing pulmonary resection and £550 per patient with pneumothorax; reducing the duration of hospitalization is considered the main savings factor.

To date, there is still little experience with the use of digital systems in the paediatric population (Table 5). The usage of digital chest drainage systems in paediatrics is poorly documented, and the articles published so far present limited conclusions as they involve few patients. Despite the small number of cases, studies in the literature have promising results:

- According to the survey conducted by Altair da Silva Costa, Jr. et al. (29), the digital drainage system facilitated decision-making in the postoperative period, thereby reducing the risk of errors in the interpretation and management of air leaks.
- The retrospective analysis of Sofia Vasconcelos-Castro et al. involved eleven patients undergoing thoracic bleb/apical pulmonary resection for primary spontaneous pneumothorax (30). Initially, patients were managed using the few existing recommendations for children, but after two cases of failure, the approach was modified by clamping the tube after a continuous air loss of 5 ml/min for at least 24 h. After changing the air loss target, the procedures were handled without complications. The algorithm suggested by the authors for the management of digital chest drainage in children consists of removing the chest tubes when the air loss is 5 ml/min for 24 h and performing chest tube clamping for a minimum of 6 h before removal.
- The consecutive prospective observational study conducted by Laura Pérez-Egido et al. included 26 patients; in 13 patients, the digital drainage system was used, and in 13 patients, the traditional drainage was used (31). The variables analysed were the duration of the drainage system, days of hospitalization, and x-rays in the immediate postoperative period correlated to the presence of the tube. According to the results obtained, the digital thoracic drainage systems provide an objective measurement of air leaks associated with an early removal of the chest tube and a reduced number of post-operative x-rays.

**Table 5:** Literature review of the pediatric population.

	Article (title, author)	Pubblication year	Journal	Number of patients
1	**An initial experience with a digital drainage system during the postoperative period of pediatric thoracic surgery;** Altair da Silva Costa, Jr, Thiago Bachichi, Caio Holanda, Luiz Augusto Lucas Martins De Rizzo	2016	Jornal Brasileiro de Pneumologia	11
2	**Digital Thoracic Drainage System: A New Tool For Pediatric Thoracic Surgery;** Sofia Vasconcelos-Castro, Mariana Borges-Dias, Miguel Soares-Oliveira	2023	Portuguese journal of cardiac thoracic and vascular surgery	11
3	**Digital thoracic drainage: a new system to monitor air leaks in pediatric population;** Laura Pérez-Egido, María Antonia García-Casillas, Isabel Simal, María Fanjul, Agustin Cañizo, Julio A. Cerdá, Beatriz Fernandez, Manuel de la Torre, Javier Ordoñez, Juan Carlos de Agustin	2018	Journal of Pediatric Surgery	26
4	Personal cases	2023		82

In our study, we compared a historical group that used traditional drainage with a group that used digital drainage. This allowed us to see a significant drop in the median number of days a chest tube reception (10.64 days in the Thopaz-treated group vs. 16.87 days in the traditional draining group) and the median number of days in the hospital (21.55 vs. 29.3). The availability of historical charts that allow one to reliably and objectively track the progress of therapy and monitor the patient's condition may have facilitated this. In addition, the new digital system provides an objective quantitative measurement of air loss and allows us to follow-up the progress of the air loss in the last 24 h, 48 h, and even 72 h, increasing confidence in making a clinical decision. Unlike analogous systems, in the absence of the possibility of recording the clinical parameters of interest over time, the decision to remove the drainage is based on what the surgeon sees at the time of the visit. We also found a decrease in postoperative x-rays (*p* < 0,05) in the digital drainage group. There were no significant differences in the incidence of postoperative complications associated with air leaks, prolonged air leaks, or pneumothorax after thoracic tube closure (*p* > 0.05). The initial learning period for using this new device is short and easy. Specialists in paediatric surgery and nurses were trained to use the device without encountering problems.

According to the doctors consulted, the main benefits obtained from the use of the new devices were:

- an easy and refined quantification of drained volumes;
- the possibility to obtain the distinction of volumes in fluids and air;
- increased mobility of the patient; Thopaz consists of a compact, lightweight, and portable aspiration unit, thus ensuring early mobilization and walking. The device guarantees a minimum autonomy of 4 h after full charging, for which it can be used at home if necessary.
- The silence of the device

On the other hand, they were disadvantages:

- The need for initial training by nursing staff;
- Lack of remote management

Nurses have preferred to use Thopaz during clinical practice to:

- Increased control of the device;
- This is a closed system in which drained fluids are collected inside a container equipped with an antibacterial filter and antivirus.
- Clear and accurate measurements of the digital system with real-time data;
- Safety during patient mobilization and during the performance of diagnostic and therapeutic procedures;
- Low-noise, quiet, and compact system.

The only disadvantage is the need for preliminary training that enables healthcare staff to manage the most potential of the device. This study showed that patients treated with Thopaz improved their ability to get out of bed and experienced the greater practicality of the system.

The present study has limitations, it is not randomized, and a multi-centre controlled studies would be needed to assess whether air flow and intrapleural pressure predict the clinical outcome of the drainage procedure and to establish guidelines for proper patient management. Despite this, the good results suggest that the use of digital chest drainage may play an increasingly central role in paediatric chest surgery, with the prospect of improving clinical outcome and optimizing care performance.

## Conclusion

The use of the digital thoracic drainage system (Thopaz) showed a reduction in the duration of the day with chest tube, post-operative length of stay, and the number of x-rays performed before removal of the device compared with the Pleur-evac system. Additional clinical benefits include objective decision-making at the time of removal of the chest tube, early mobilization of the patient, and reduction of radiation exposure. From the results described above, the use of Thopaz in the paediatric population seems to be safe and potentially beneficial; we therefore believe that this digital drainage system can become a very useful tool, if not indispensable, in the management of the patient undergoing chest surgery.

## Data Availability

The original contributions presented in the study are included in the article/Supplementary Material, further inquiries can be directed to the corresponding author.
